# A New Method of Removing Nasal Polypi

**Published:** 1884-09

**Authors:** 


### A New Method of Removing Nasal Polypi.

Dr. William Ralph Bell says: "I take the liberty of bringing the mode of treatment before the notice of your readers which I have practiced with the very best results in several cases. It obviates any trouble from hemorrhage, which is frequently the case when the forceps or hooks is used; it is painless and very simple. I get my patient to blow strongly through the affected nostril, closing the other with his finger. The polypus will be brought down so that it can be easily seen through the external nares; then with my hypodermic syringe charged with a solution of tannic acid in water (of the strength of twenty grains to the fluid drachm), I pierce the polypus with the needle, and inject ten, fifteen, or twenty minims of solution, according to size of tumor. In a few days the polypus shrivels and dries up (tanned); it comes away without any trouble or pain, and looks like a clot of dry blood, my patients usually removing it by blowing the nose or by their fingers. In only one case, that of an old lady, had I occasion to remove it myself, and in her case I think she was afraid to do so, for when I seized it with dressing forceps, I required to make no traction to bring it away."—*Canada Med. Record.*

